# Fabrication and characterization of resistive double square loop arrays for ultra-wide bandwidth microwave absorption

**DOI:** 10.1038/s41598-021-91868-y

**Published:** 2021-06-17

**Authors:** Ji-Young Jeong, Je-Ryung Lee, Hyeonjin Park, Joonkyo Jung, Doo-Sun Choi, Eun-chae Jeon, Jonghwa Shin, Jun Sae Han, Tae-Jin Je

**Affiliations:** 1 Department of Nano Mechatronics Engineering, University of Science and Technology (UST), Daejeon, 34113 Republic of Korea; 2 Department of Nano Manufacturing Technology, Korea Institute of Machinery and Materials (KIMM), Daejeon, 34103 Republic of Korea; 3 Advanced Cutting Tools Research Team, Daegu Mechatronics and Materials Institute (DMI), Daegu, 42714 Republic of Korea; 4 Department of Materials Science and Engineering, Korea Advanced Institute of Science and Technology (KAIST), Daejeon, 34141 Republic of Korea; 5 School of Materials Science and Engineering, University of Ulsan, Ulsan, 44610 Republic of Korea

**Keywords:** Engineering, Materials science

## Abstract

Microwave absorbers using conductive ink are generally fabricated by printing an array pattern on a substrate to generate electromagnetic fields. However, screen printing processes are difficult to vary the sheet resistance values for different regions of the pattern on the same layer, because the printing process deposits materials at the same height over the entire surface of substrate. In this study, a promising manufacturing process was suggested for engraved resistive double square loop arrays with ultra-wide bandwidth microwave. The developed manufacturing process consists of a micro-end-milling, inking, and planing processes. A 144-number of double square loop array was precisely machined on a polymethyl methacrylate workpiece with the micro-end-milling process. After engraving array structures, the machined surface was completely covered with the developed conductive carbon ink with a sheet resistance of 15 Ω/sq. It was cured at room temperature. Excluding the ink that filled the machined double square loop array, overflowed ink was removed with the planing process to achieve full filled and isolated resistive array patterns. The fabricated microwave absorber showed a small radar cross-section with reflectance less than − 10 dB in the frequency band range of 8.0–14.6 GHz.

## Introduction

Recently, with increasing importance of electronic warfare in the military field, microwave absorbers with perfect absorption against incident electromagnetic waves are vigorously studied. The conventional method for absorbing a microwave is using a paint that contains tiny spheres made of carbonyl iron or ferrite. These particles convert microwave energy to heat through coupled electromagnetic oscillations^1–4^. However, the required minimum thickness of paint for a high absorptance and low reflectance is considerably high. Because ferrite materials have a high specific gravity, thick ferrite paints can increase the weight of the applied body significantly. For this reason, light and thin absorbers that can strongly absorb microwave without using heavy ferrite materials are in demand. Such needs have resulted in various research on ‘electromagnetic metamaterial absorbers’.

Electromagnetic metamaterials can possess unique electromagnetic attributes that can be explained with unusual values of effective material parameters such as magnetic permeability and electric permittivity. Smith, Padilla, and others have proposed that a broad range of electromagnetic responses can be achieved by arranging conductive scatterers^5,6^. Based on their research, many studies have been actively conducted for unnatural properties such as negative refractive index^7,8^, super resolution^9,10^, and wave absorption^11–13^. Especially, when applied to absorbers, electromagnetic metamaterials can improve the device performance and impart desirable qualities such as polarization-insensitivity, wide incident angle characteristics, multi-resonances, and multi-band absorption^14–16^. Due to the vastly increased freedom of material choice, electromagnetic metamaterial-based absorbers can have additional advantages in strength, elasticity, heat conduction, and other mechanical or chemical properties. However, there are still hurdles remaining in implementing these ideas in real devices and finding fabrication-friendly designs as well as practical and cost-effective fabrication methods is among the missing pieces. For example, the lumped resistor inclusions in some of the proposed metamaterial absorber designs are not ideal in terms of fabrication cost and durability. To solve these problems, many studied metamaterials using a polymeric conductive material including carbon or graphite with periodic patterns as a circuit and specific sheet resistance instead of using materials having lumped resistances^14,17–20^.

Fabrication of microwave metamaterials composed of conductive materials usually involve a screen-printing process or an inkjet printing process^20–25^. These processes can fabricate various shapes of circuit patterns (square loop^26–28^, cross dipole^29–31^, fivefold^23,25,32^, square patch^33–35^, and so on) on a dielectric substrate. Although printing processes are promising candidates for low-cost fabrication of microwave metamaterials, it is difficult to adjust the sheet resistance value for different parts of a pattern in a finely-controlled fashion. In addition, typical printing processes result in embossed (rather than engraved) patterns because they are additive process depositing new material on flat substrates. The embossed conductive materials are more prone to mechanical deformation or damage in the removing and curing process of the ink^36^. By contrast, engraved ink patterns on mechanical machined substrates allow a fine control of the thicknesses of different parts of the pattern on the same layer simply by controlling the machining depth. Moreover, rheology properties of inks have much less effects on final shapes of patterns because inks only fill engraved patterns.

The aim of this study was to develop and characterize the manufacturing process for a microwave absorber with resistive double square loop arrays. The developed process consisted of micro-end-milling, inking, and planing steps. End-milling means engraving double square loop arrays. Inking is filling conductive carbon ink to the engraved array structures. Planing is removing overfilled conductive ink and machining the planar surface. Under the established process, the fabricated microwave absorber having a thickness of 4.16 mm showed RCS reduction with reflectance less than − 10 dB in the range of 8.0–14.6 GHz.

## Experiments

### Design of the microwave absorber

A typical metamaterial absorber is composed of a metal-backed dielectric layer and a top resistive layer. The impedance of the metal-backed substrate can be calculated with the following equation:

1 $${\eta }_{subs}=i\frac{{\eta }_{0}}{n}\mathrm{tan}(n{k}_{0}d)$$

where n is the refractive index, ${\eta }_{0}$ is the impedance of free space (air), d is the thickness of the non-magnetic dielectric, and ${k}_{0}$ is the wave number in free space. With the concept of equivalent circuit model, the total impedance value of the whole absorber structure can be calculated with the following equation:

2 $$\frac{1}{\eta }=\frac{1}{{\eta }_{subs}}+\frac{1}{{\eta }_{r}}$$

where $\eta$ is the impedance of the whole absorber structure and ${\eta }_{r}$ is the impedance of the top resistive layer. The required impedance value of the top resistive layer can be calculated for the specific frequency range with the predetermined value of thickness and the dielectric index. The strategy to attain the required impedance value is very wide. Various designs including cross, ring, and patch for the resistive layer have been reported. We selected double square loop arrays and designed to have different sheet resistance for each square loop in order to confirm the performance of the developed manufacturing process due to high degrees of freedom in its shape, including length, width, and depth of each loop.

**Figure 1:**
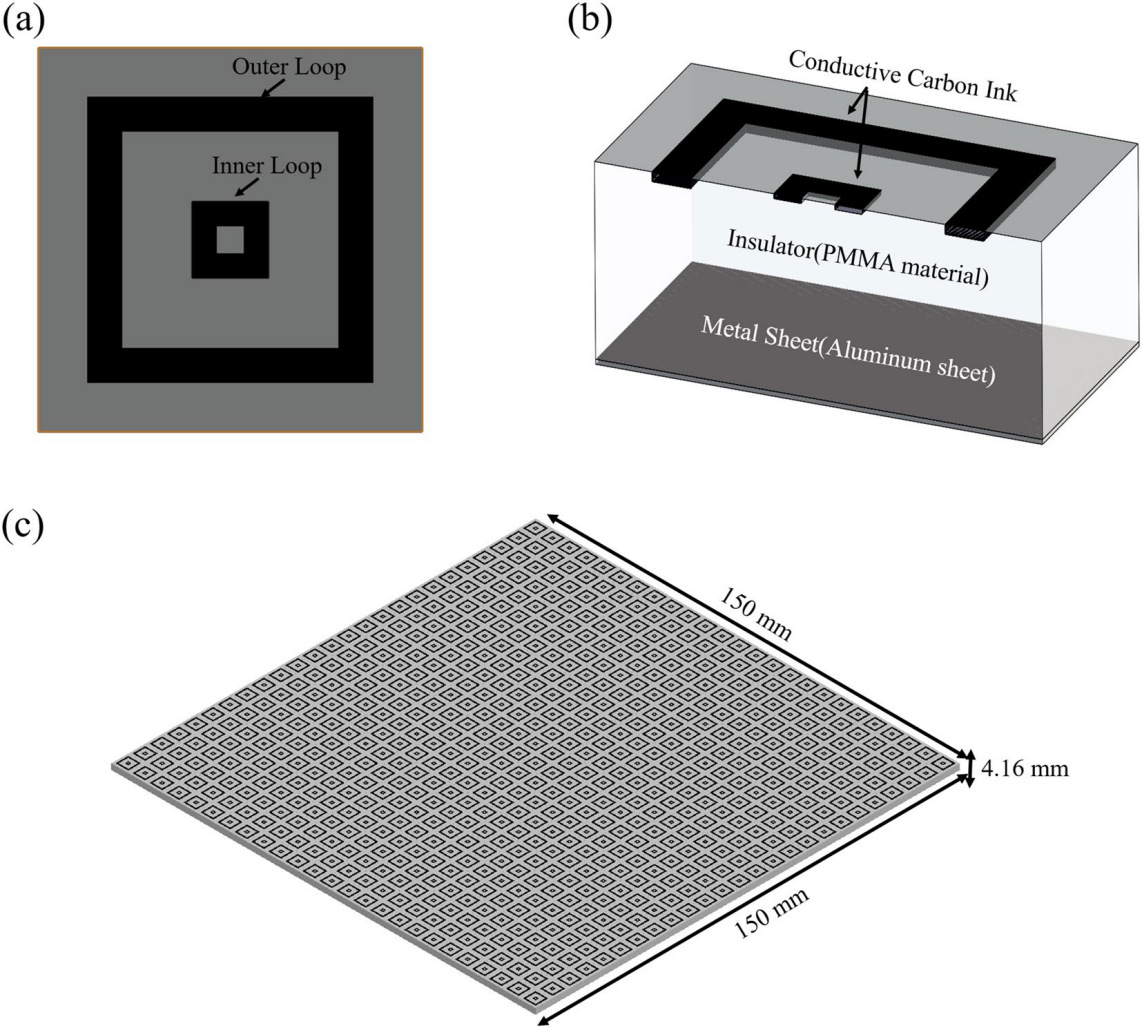
Structural design of the microwave absorber with resistive double square loop arrays. (**a**) Top view of unit-cell, (**b**) cross-section of the unit-cell, (**c**) full structure of the microwave absorber.

Figure 1a,b show a designed unit cell of microwave absorber. This absorber structure is optimized with a PSO (particle swarm optimization) algorithm applied to a Lumerical FDTD solver. The target performance of the absorber was set to be lower than 20% reflectance at 5–14 GHz, a sufficient performance to confirm the effectiveness of the manufacturing process developed in this study. Eight parameters were optimized during the process, including period, thickness of the structure, length, width, depth of each ring with a predetermined refractive index of substrate $n=1.732+0.00866i$ and an ink conductivity $\sigma =2352.9\,{\mathrm{S}}/{\mathrm{m}}$*.*

The microwave absorber was designed to have three layers with resistive patterns, insulator material, and a metal sheet: (i) conductive carbon ink based on frequency selective surface layer, (ii) PMMA based insulating layer for the spacer to adjust the resonance position between incident microwave from radar and reflected microwave from ground, and (iii) metal sheet layer for reflection of transmitted microwave. This absorber material had a 144-number of double square loops in 150 mm^2^ area of PMMA material with a thickness of 4.16 mm as shown in Fig. 1c.

**Figure 2:**
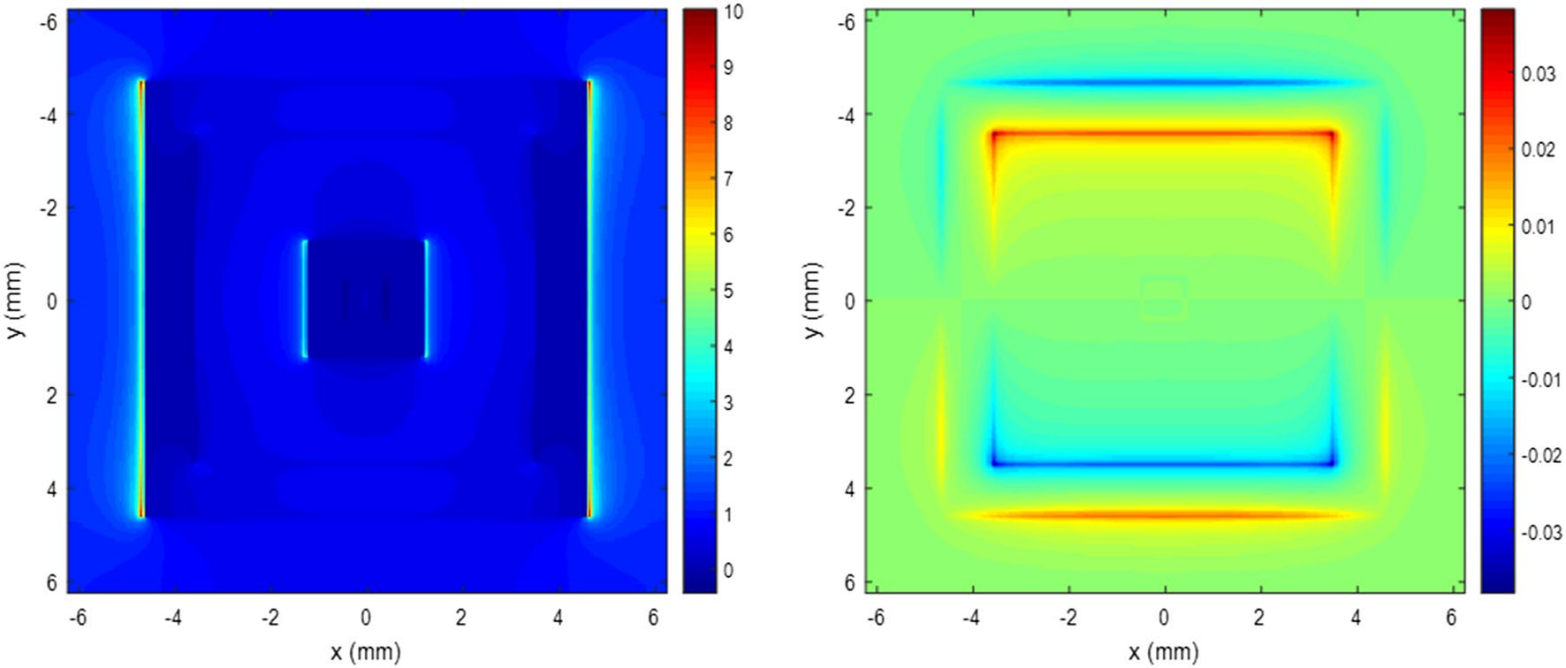
Electric and magnetic field distributions of a double square loop pattern at 5.7 GHz.

**Figure 3:**
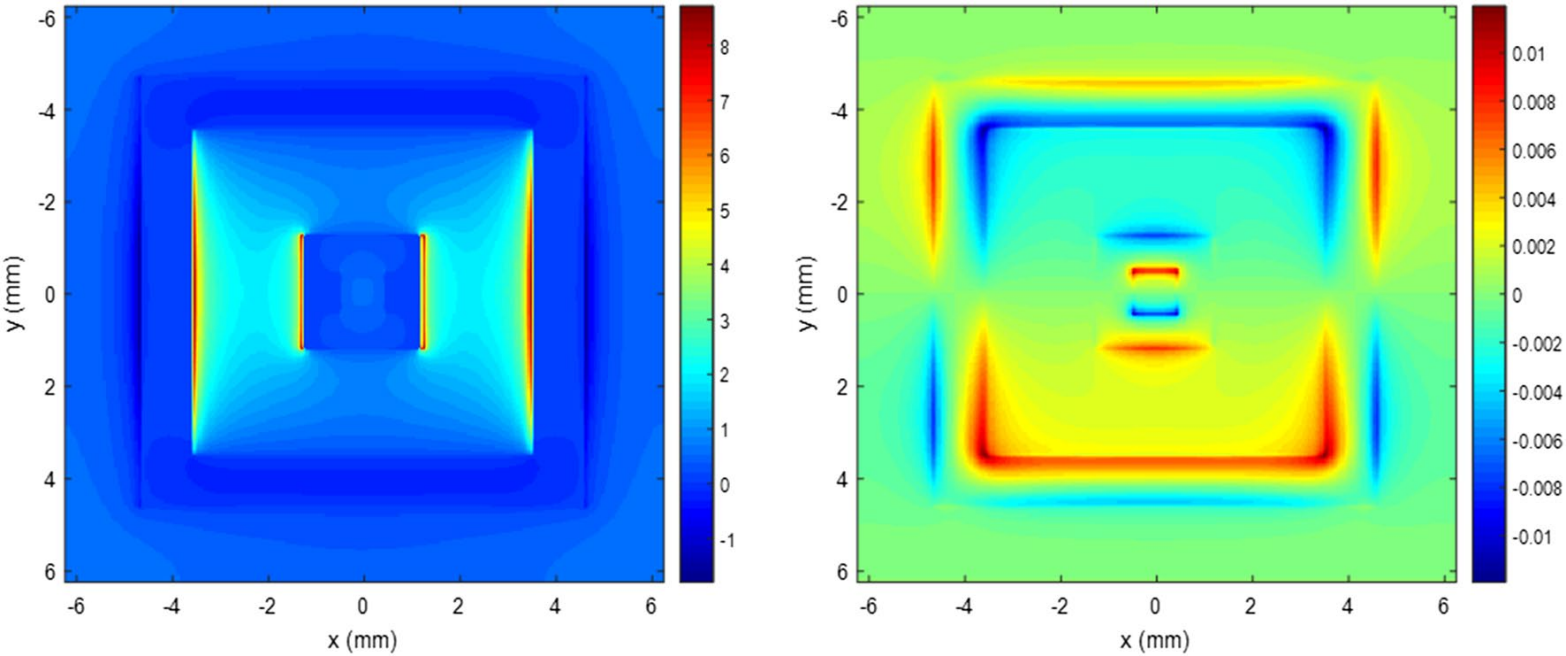
Electric and magnetic field distributions of a double square loop pattern at 12.7 GHz.

**Figure 4:**
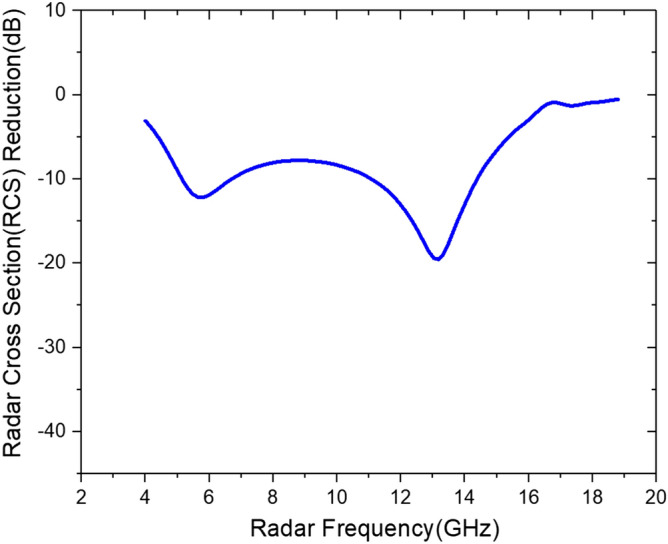
Simulated RCS reduction of the designed microwave absorber at 4 to 18 GHz.

Figures 2 and 3 show the real part of the E_x_ field and Hz field distributions at the middle plane of the double square loop. In the simulation settings, a plane wave was normally incident on the absorber structure and the electric field was on the X direction. Figure 2 shows electric and magnetic field distributions for 5.7 GHz wave incidence. Figure 3 shows electric and magnetic field distributions for 12.7 GHz where the reflectance dips occur. As shown in each figure, two sides of the ring parallel to the external electric field showed inductive behaviors while others showed capacitive behaviors. Due to these resonant behaviors of conductive rings at different frequencies, high absorption can occur. Simulation results for reflectance of the absorber structure are shown in Fig. 4. The designed structure showed an absorption performance more than 80% at 4.7–14.3 GHz.

### Manufacturing process of the microwave absorber

**Figure 5:**
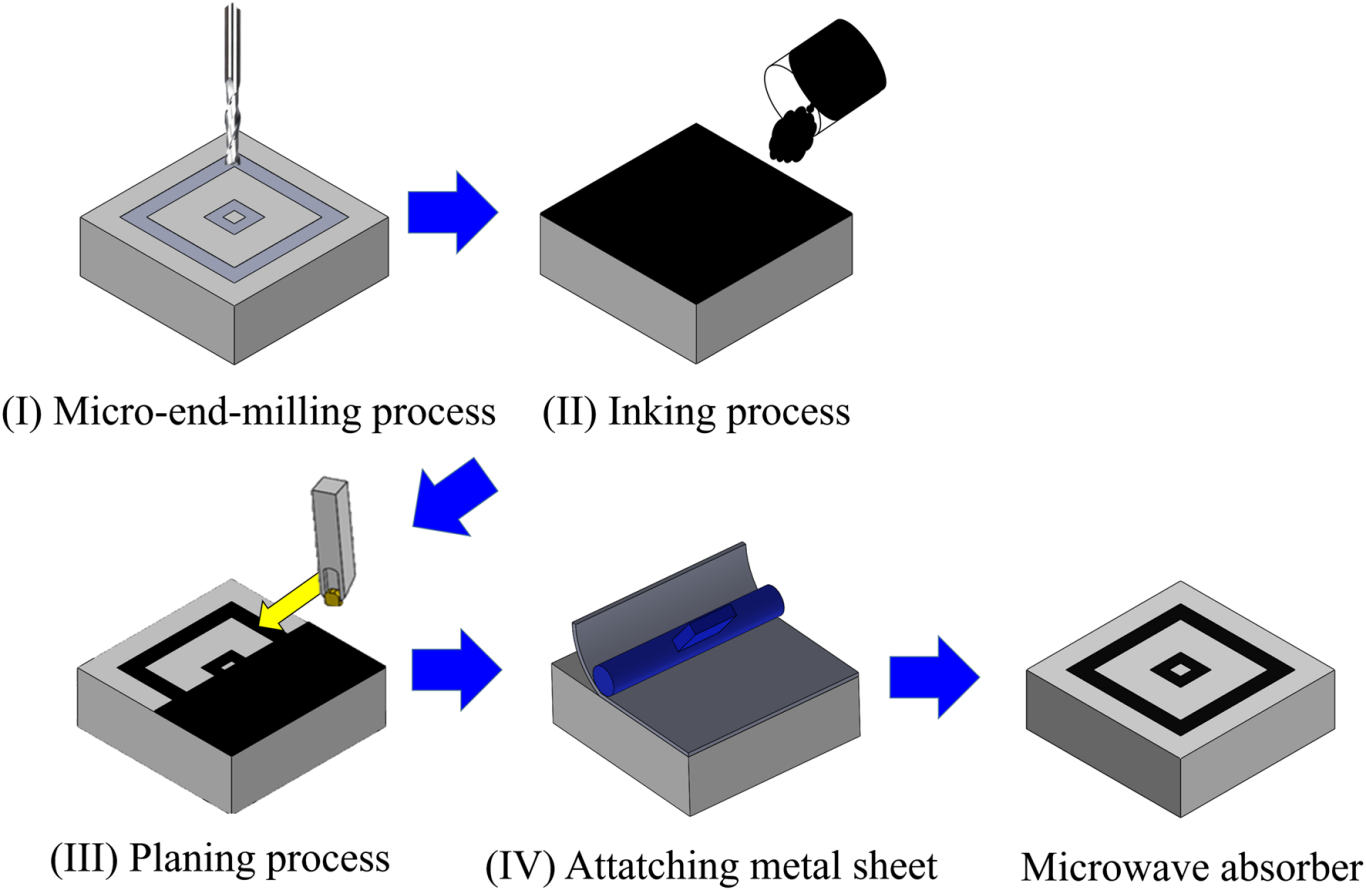
A schematic diagram showing the developed manufacturing processes for microwave absorber with resistive double square loop on a PMMA.

Figure 5 shows the developed manufacturing process for the designed microwave absorber with resistive double square loop arrays on an insulator material. The developed process had four steps. The first step was a micro-end-milling process for engraving array structure on the surface of the insulator layer. The second step was an inking process for fill up the engraved array structure with the conductive ink. The third step was a planing process to remove the conductive ink overfilled in excess of the array structure. The final step was attaching a metal sheet to the bottom of the insulator layer. In this study, the PMMA material was used as an insulator layer. This is because it has advantages such as good machinability, appropriate refractive index, and superior transparency. The refractive index of PMMA was confirmed by simulation of magnetic and electric filed distributions. Transparency of PMMA has advantages in confirming the residual conductive carbon ink on the microwave absorber after removing overfilled conductive ink by a planing process. For these reasons, the PMMA was chosen as the insulator material in this study. In the entire step, each processing condition was experimentally developed and optimized.

## Results and discussion

### Micro-end-milling process

**Figure 6:**
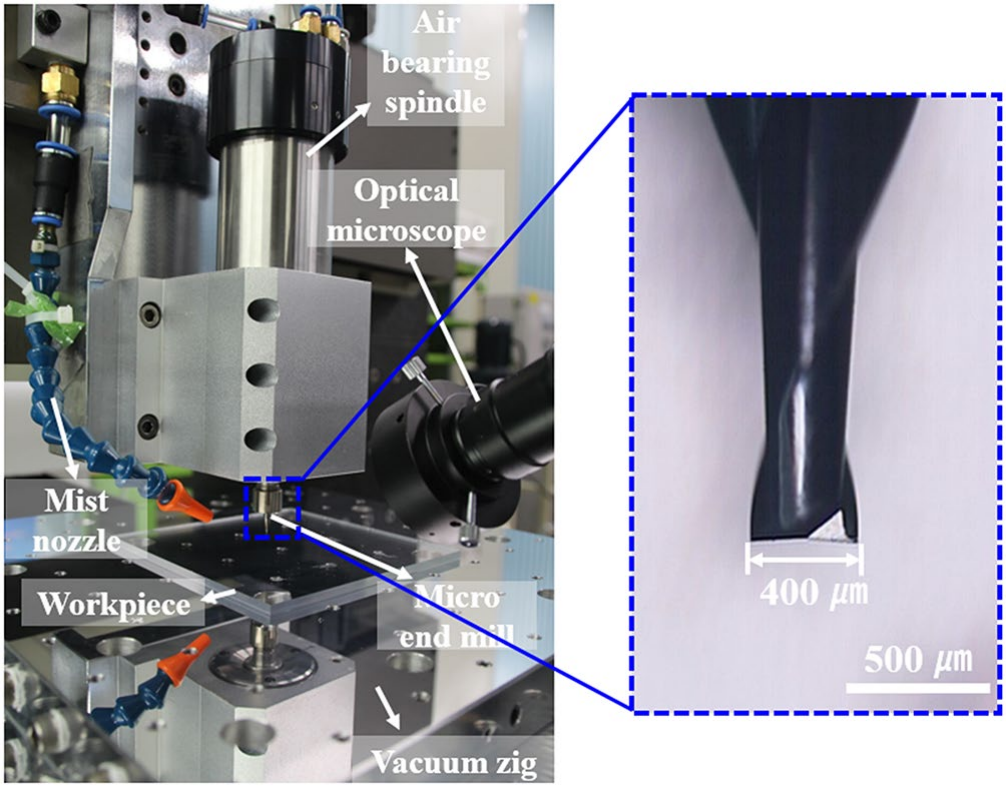
The micro-end-milling system for engraving of double square loop arrays on the PMMA workpiece and the cutting tool of TiAlN coated solid carbide.

As the first step, a flat PMMA workpiece was mechanically machined using an ultra-precision micro-end-milling system. Figure 6 shows an ultra-precision machining system having a three-axis with 10 nm positioning resolution and air bearing spindle having a max rotation speed of 100,000 rpm. TiAlN (Titanium Aluminum Nitride) coated solid carbide with two flutes and a square end shape having a diameter of 400 μm was used as a cutting tool for strong wear resistance and lubrication ability. Cutting oil of ISOPAR-H was spread to the endmill using a mist nozzle to prevent built-up edge caused by the characteristic of PMMA that could easily stick to the cutting tool surface because of cutting heat.

Optimized machining conditions are summarized in Table 1. Since the machining quality by the end-milling process can greatly affect the performance of microwave absorption, quality optimization of roughness, shape, and size accuracy is needed by optimizing machining conditions. Especially, burr has a significantly degrading quality of roughness and shape accuracy of the engraved pattern. Burr can be easily formed on the edge and machined surface in the first and finish cuts. Moreover, the volume of burr tends to increase as more materials are removed at one time^37^. For these reasons, in order to minimize burr formation, the depth of cut was set to be 5 μm for the first and finish cuts. The rough cut with a relatively low impact on the machining quality was applied to the remaining depth. Total cutting depths of each pattern were designed at 22 μm for the inner pattern and 30 μm for the outer pattern that included 5 μm of the planing margin. Theoretical surface roughness after the end-milling process can be calculated with Eq. (3)^38^.

3 $${R}_{a,e}=\frac{{{f}_{t}}^{2}}{32\times (D/2\pm {f}_{t}\times {n}_{t}/\pi )}$$

where *R*_*a,e*_ is the average surface roughness machined by the micro-end-milling process, *f*_*t*_ is the feed per revolution, *n*_*t*_ is the number of teeth on the end-mill, and *D* is edge diameter of the tool. The plus sign means an up-milling process and the minus sign means a down-milling process. The up-milling process was applied to obtain excellent surface roughness as minimizing burr formation. The rotation speed of the cutting tool and feed per revolution were applied to 80,000 rpm and 9 μm/rev, respectively. The theoretical surface roughness under the above conditions was calculated to be about 12.3 nm.

**Table 1:** Optimized machining conditions of the micro-end-milling process for double square array patterns on the PMMA surface.

Cutting tool rotation speed (rpm)	80,000				
Feed per revolution (μm/rev)	9				
Cutting depth (μm)		First cut	Rough cut	Finish cut	Total
	Inner pattern	5	12	5	22
	Outer pattern	5	20	5	30

**Figure 7:**
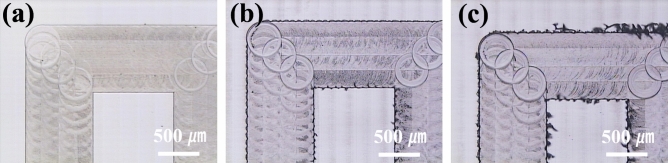
Microscope images of the machined inner pattern: (**a**) the first inner pattern, (**b**) the 72nd inner pattern, and (**c**) the 144th inner pattern.

Figure 7a,b,c show the first, 72nd, and 144th inner patterns of double square loops after the end-milling process, respectively. The first inner pattern showed clear edges with a smoothly machined surface. However, with increasing machining number of patterns, the size of the burr was also increased and the machined surface was roughened with a significant tool mark.

**Figure 8:**
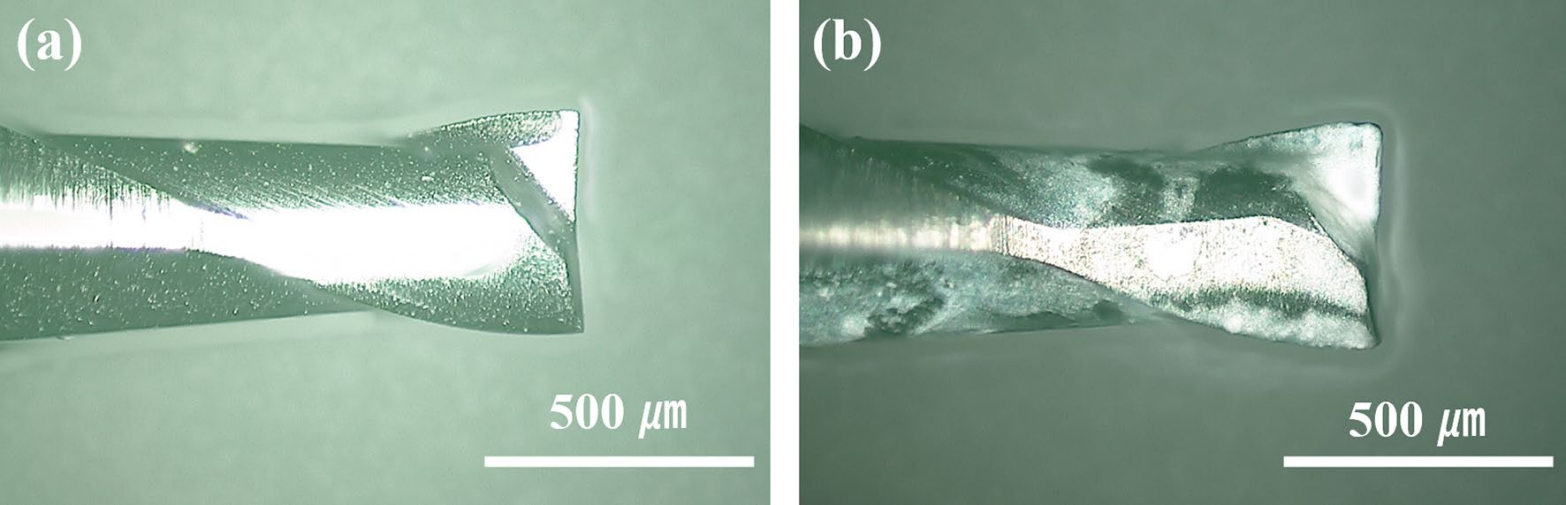
Microscope images of cutting tools before and after machining: (**a**) before machining and (**b**) after machining of the 144th double square loop.

Figure 8a,b show cutting tools before and after machining of the 144th double square loop. Machinability of the cutting tool was remarkably decreased because the machined materials were adhered to the cutting edge. An ultrasonic cleaning method was additionally applied for removing the built-up edge on the cutting tool. The cleaning time was set to be 3 min after machining each of 24 patterns.

**Figure 9:**
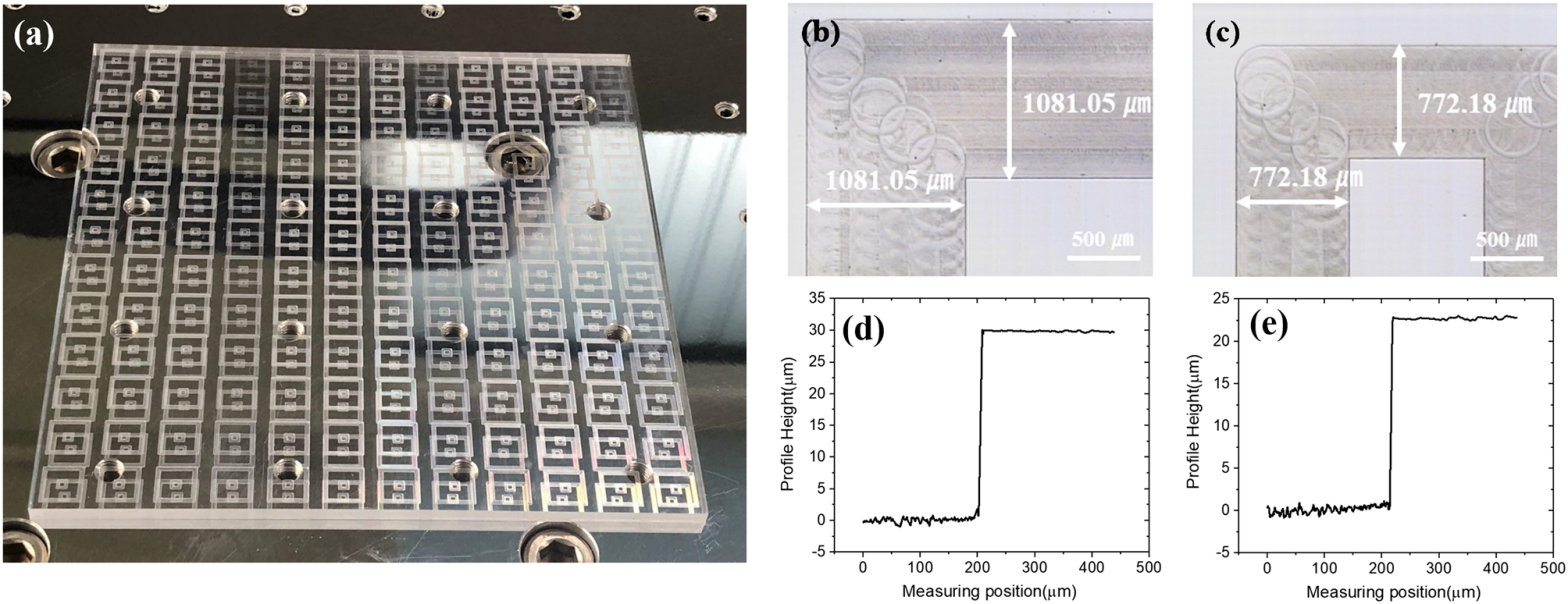
Measuring results of engraved double square loop on the PMMA workpiece by the micro-end-milling. (**a**) A camera image of machined PMMA workpiece after the end-milling process, (**b**) top view of the outer pattern, (**c**) top view of the inner pattern, (**d**) depth profile of the outer pattern, and (**e**) depth profile of the inner pattern.

Figure 9 shows machined PMMA workpiece with 144-member double square patterns (150 mm × 150 mm in width and 4.05 mm in thickness). Widths of the machined inner and outer patterns were about 772.18 μm and 1081.05 μm, respectively. Heights of the machined inner and outer patterns were about 22.05 μm and 30.35 μm, respectively. Their average surface roughness was about 55 nm. The surface roughness was larger than twice of theoretical value because factors such as vibration and machining environment were excluded from the theoretical formula. In consideration of these factors, we fabricated an excellent surface with an average roughness of less than 100 nm. Also, machined patterns were successfully fabricated within 1% of machining error compared to the target size through an optimized end-milling process.

### Inking and planing processes

As a second step, machined double square loop arrays on the surface of PMMA were filled with conductive carbon ink by the inking process. The most important factor in the inking process was the sheet resistance of conductive carbon ink. If the sheet resistance was different from the target value for generating electromagnetic field, the bandwidth of radar absorption could be changed or the performance of the RCS reduction could be degraded. The sheet resistance of the conductive carbon ink used in this experiment was controlled by mixing polymers of conventional carbon ink and silver paste with a solvent of thinner. Properties of the conductive carbon ink, the silver paste, and the developed conductive ink are presented in Table 2. The conductive carbon ink was GST 4500 manufactured by SunChemical Co. in the USA. It had a sheet resistance of 50 Ω/sq at 25 μm. The silver paste was p-100 manufactured by CANS Co. in JAPAN. It had a sheet resistance of 4 × 10^–8^ Ω/sq at 25 μm. In order to achieve designed sheet resistance, conductive ink, silver paste, and thinner were mixed as a ratio of 47.5:1:1. The mixed conductive carbon ink had sheet resistance of 15 Ω/sq at 25 μm.

**Table 2:** Properties of the conductive carbon ink and the silver paste.

Type	Pigment	Solid ratio (%)	Viscosity (Poise/25 ℃)	Sheet resistivity (Ω/sq at thickness of 25 μm)	Manufacturer and model name
Carbon ink	Carbon, graphite	40–43	85–105	50	SunChemical GST4500
Silver paste	Silver	–	230	4 × 10^–8^	CANS p–100

For filling the ink, the machined PMMA workpiece was detached from the machining table of the micro-milling system. The machined PMMA surface was then completely covered by manually pouring and spreading the developed conductive ink onto its surface. To completely cure the ink without deforming the workpiece by heating, the conductive ink was cured at room temperature (23℃) for one day. The workpiece with the fully cured ink was remounted on the planing system that changed the cutting tool from micro-end-mill to single crystal diamond with a nose radius of 10 mm.

**Figure 10:**
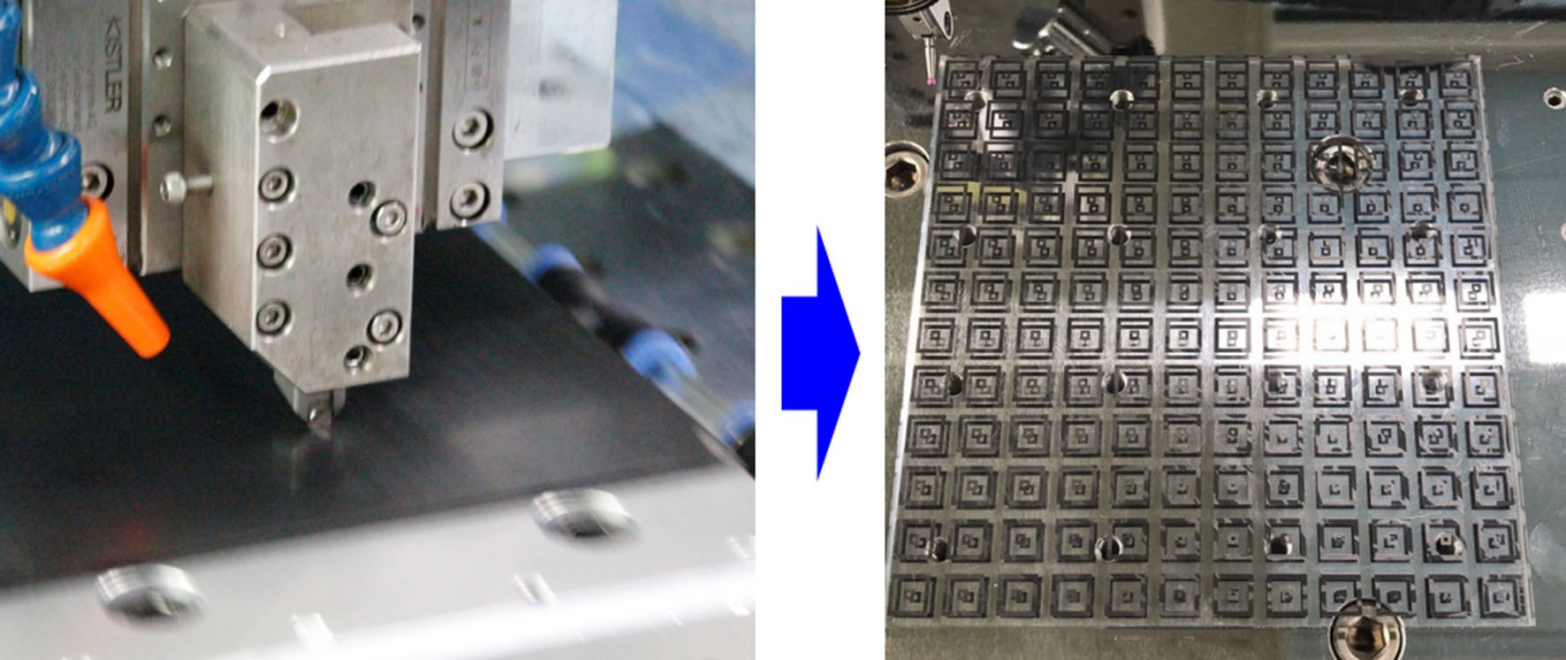
The planing process for removing the conductive carbon ink applied over the PMMA workpiece and the machined workpiece after removing the over filled ink.

In the third step, the over filled ink was removed using the planing process to completely isolate each pattern. In the planing process, the theoretical surface roughness was calculated with Eq. (4)^39^.

4 $${R}_{a,p}=\frac{0.30321\times {f}^{2}}{R/2}$$

where *R*_*a,p*_ is the average surface roughness machined by the planing process, *f* is the cutting pitch, and *R* is the diameter of nose radius of the cutting tool. According to this equation, the roughness of the machined surface by the planing process was determined by the nose radius of the cutting tool and the cutting pitch. Since the nose radius of the cutting tool was a fixed variable, the cutting pitch was set to be 50 μm to have a theoretical surface roughness of 31.25 nm. Figure 10 shows the planing process to remove the overfilled ink on the PMMA workpiece. The overfilled ink was removed step by step as cutting depth was decreased from the highest position to the target thickness of 4 mm.

**Figure 11:**
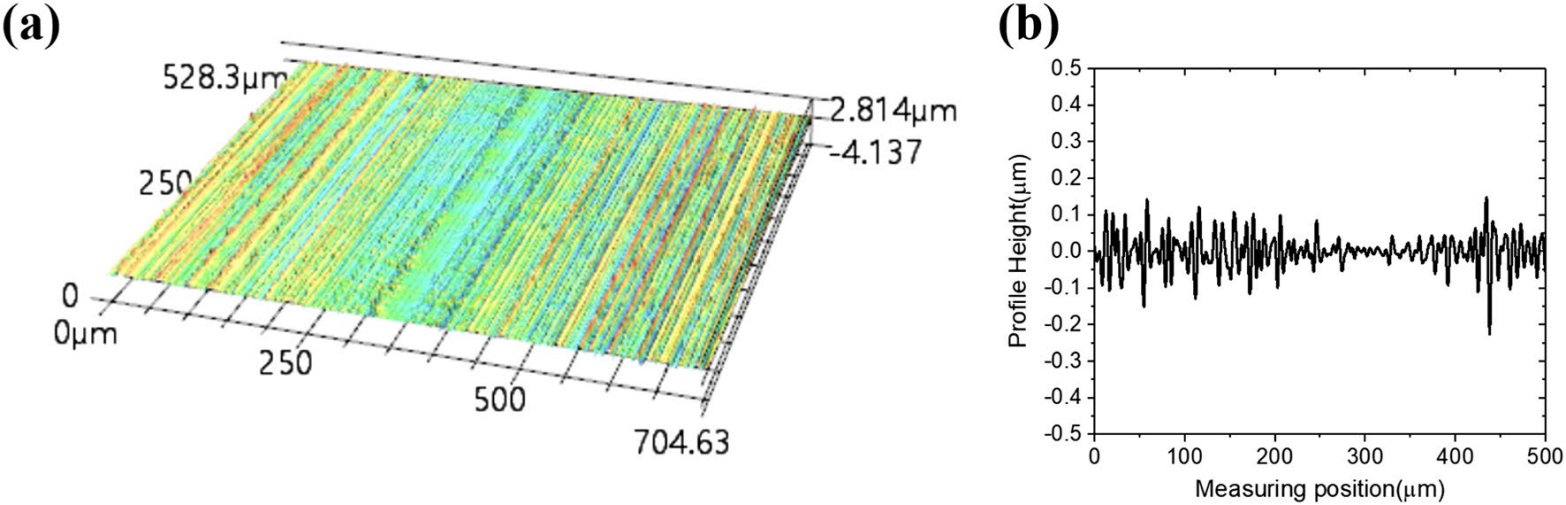
Confocal laser microscope measuring results of the PMMA surface after removing the overflowed conductive ink with the planing process. (**a**) 3D image of the PMMA surface after removing the overfilled conductive ink, (**b**) roughness profile of the PMMA surface after removing the overflowed conductive ink.

**Figure 12:**
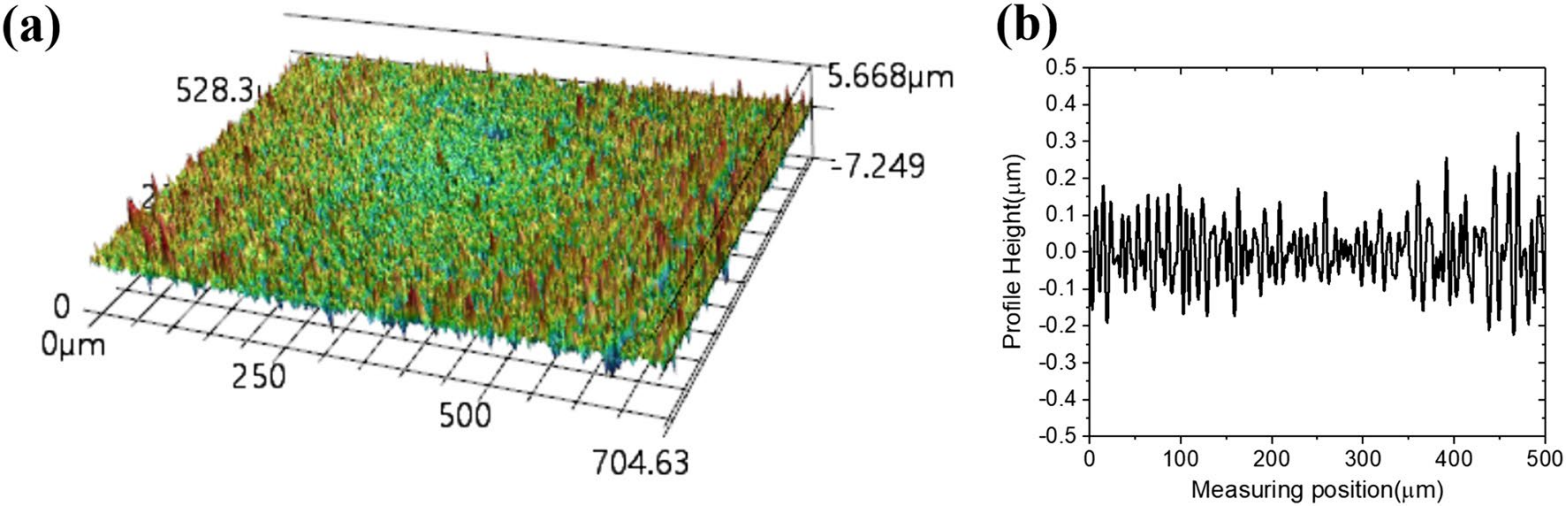
Confocal laser microscope measuring results of the resistive double square loops filled with the conductive ink machined by the planing process. (**a**) 3D image of ink surface after removing the overflowed conductive ink, (**b**) roughness profile of the ink surface after removing the overflowed conductive ink.

The machined surface by the planing process was measured using a confocal laser microscope (Keyence VK-X1000). Figure 11a shows a 3D image of the PMMA surface after removing overfilled ink. Linear lines were formed on the PMMA surface with an equal direction to the cutting direction. Figure 11b shows roughness profile of this surface. *R*_*a,p*_ is 38 nm which is close to the calculated value with Eq. (4). Figure 12a shows a 3D image of the carbon ink surface after removing the overfilled ink. Unlike the PMMA surface, the cured ink surface machined by the planing process was difficult to identify cutting lines. It was rougher than the PMMA surface. The *R*_*a,p*_ of this surface was 68 nm as shown in Fig. 12b, which was close to twice of the value shown in Fig. 11b. This result was caused by the fact that the perfectly cured ink was more fragile than the PMMA. It was also caused by the irregular surface structure because of mixing various materials. Although the machined surface of PMMA and the surface of the conductive ink were different, both surfaces showed excellent characteristics with an average roughness of less than 100 nm, which had less effect on the progress of microwaves. In addition, each array was completely filled and isolated without showing any residual ink on the surface. After the planing process as the final step, an absorption material for ultra-wide bandwidth microwave from C- to Ku-band (about 5–15 GHz) was fabricated by attaching a 160 μm of the adhesive-applied aluminum sheet as a reflection layer on the back surface of PMMA workpiece for oscillating of the microwave.

### Characterization of the microwave absorber with resistive double square loop arrays

**Figure 13:**
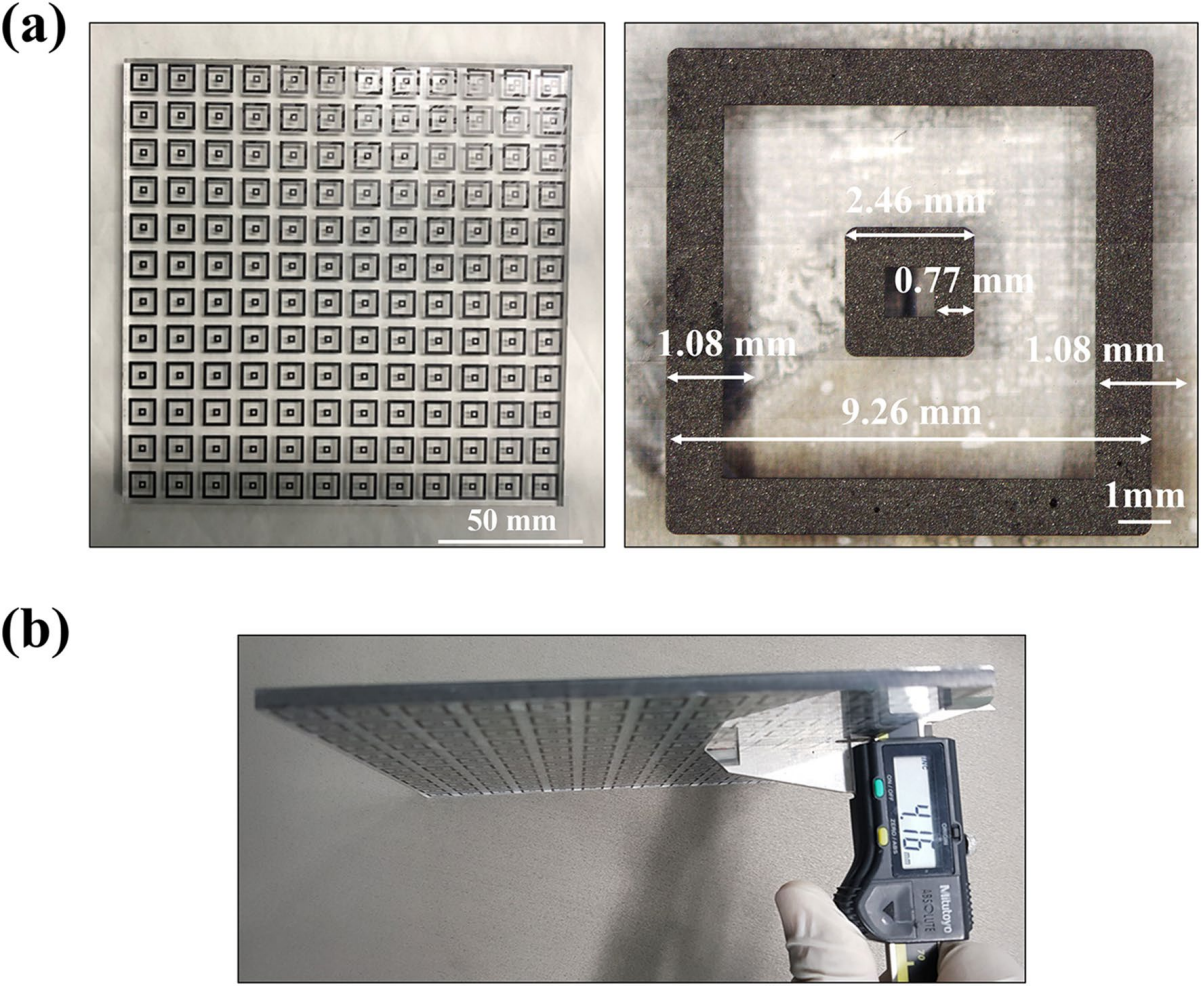
A microwave absorber fabricated by the developed manufacturing process. (**a**) Top view of the fabricated microwave absorber and enlarged image of resistive double square loop pattern with measuring the size and width of the pattern, (**b**) side view and measuring thickness of the microwave absorber.

Figure 13 shows fabricated microwave absorber with resistive double square loop arrays by the manufacturing process developed in this study. Figure 13a shows the fabricated microwave absorber with an area of 150 mm^2^ and an enlarged view of a double square pattern which has fully filled and cured conductive ink. Figure 13b shows the total thickness of 4.16 mm that comprises two polymer materials and a metal sheet. The PMMA material with a thickness of 4 mm conducts an isolation layer between the electromagnetic field and the ground. The resistive double square loops with array numbers of 12 × 12 generate an electromagnetic field with a distance of 4 mm from the ground surface. The size and the width of the inner pattern were about 2.46 mm and 0.77 mm, respectively. The outer pattern size and width were about 9.26 mm and about 1.08 mm, respectively. These patterns were machined within a size error of 1% compared to the target size and width. An aluminum sheet with a thickness of 0.16 mm reflected transmitted microwaves.

**Figure 14:**
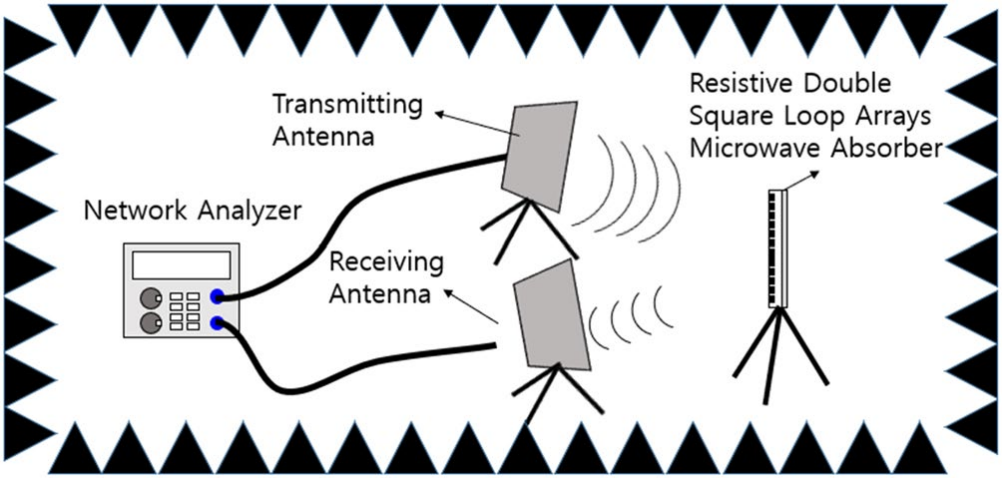
Schematic layout of the RCS reduction measurement system.

To characterize absorption properties, an RCS-measurement setup was constructed as shown in Fig. 14. A transmitting antenna with a focusing lens launched a Gaussian-like microwave beam to the target. A receiving antenna, also with a focusing lens, collected the reflected microwave from the target. These antennas were connected to a network analyzer. To evaluate the RCS reduction characteristics over a wide bandwidth, two sets of horn antennas, rated at 8.0–12.5 GHz and 12.7–18.0 GHz, respectively, were used.

**Figure 15:**
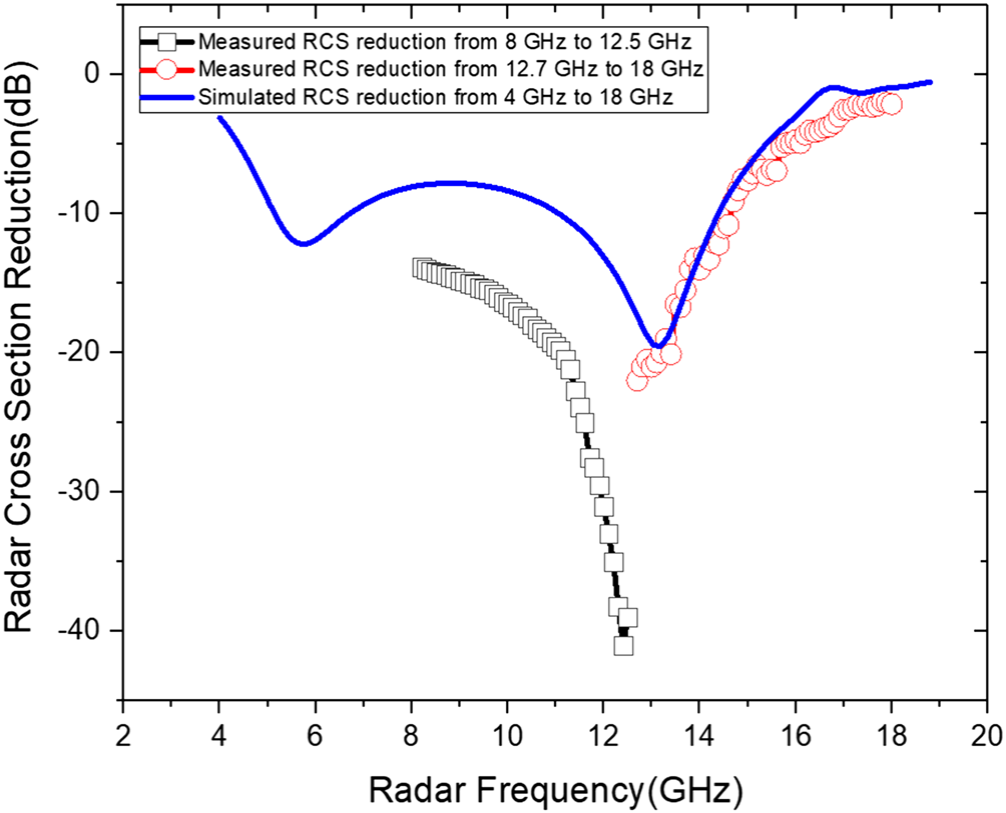
Measured and simulated RCS reduction of the fabricated microwave absorber according to radar frequency.

Figure 15 shows the measured reflectance as a function of the microwave frequency. The microwave absorbing performance of the fabricated microwave absorber was the highest near the center of the measured frequency range and gradually decreased towards the boundary. In the frequency band of 8.0–14.6 GHz, the measured reflectance was less than − 10 dB. The minimum reflectance of − 41 dB was measured at 12.4 GHz. From simulations, it is expected that the frequency range with more than 10 dB attenuation may extend down to ~ 5 GHz, but it requires further investigation. Although the measured RCS reduction performance showed a slight difference compared to simulated results, the tendency of absorption performance was similar. The cause of such difference between simulated and measured such as the maximum RCS reduction value and the resonance frequency was attributed to the non-uniformity of the applied conductive ink. This problem can be improved through a future study by optimizing the conductive ink application and curing process.

Table 3 shows results of comparison in terms of layer number, pattern shape, thickness, bandwidth, and the maximum RCS reduction with other state-of-art broadband microwave absorbers. Previously reported microwave absorbers having multiple layers of resistive patterns have different sheet resistances or shapes on each layer to increase the absorbing performance and broaden the effective frequency range. On the other hand, the microwave absorbers by the proposed manufacturing process in this study can have patterns with different sheet resistances on a single layer. This advantage allows, thinner absorber designs with good absorption performance.

**Table 3:** Comparison of layer number, pattern shape, thickness, bandwidth, and the maximum RCS reduction with other state-of-art broadband microwave absorbers.

Ref.	Manufacturing process (conductive material)	Pattern shape	Number of patterned layers	Thickness (mm)	Bandwidth (GHz)	Maximum RCS reduction (dB)
^40^	Printing process (carbon ink)	Square loop array	2	6.4	5–30.8	− 42
^41^	Etching (copper)	Double square loop	1	4.3	0.9–1.8	− 41
^42^	(Carbon ink)	Square array	2	3.5	8–18	− 27.5
^43^	Silk printing (carbon ink)	Square array	2	7.6	5.7–18	− 49
^23^	Inkjet printing (CNT ink)	Jerusalem Cross + mesh grid	2	5.1	8.2–44	− 43
This work	Mechanical machining process (carbon ink)	Double square loop	1	4.2	8–14.6	− 41

## Conclusion

In this study, a manufacturing process was developed for a wide-bandwidth microwave absorber. The fabricated microwave absorber has 144 resistive double square patterns without connection among them. These patterns were precisely fabricated within a size error of 1% and a flat surface with an average roughness under 100 nm. The developed microwave absorber showed a performance of RCS reduction with reflectance less than − 10 dB over 8 to 14.6 GHz frequency range. To extend the RCS reduction performance to the entire Ku-band or higher frequencies and to make the performance more uniform across the frequency band, shapes of resistive array patterns and properties of the conductive carbon ink need to be optimized with further study.
